# Supplementary material for: Epidermal retinol dehydrogenases cyclically regulate stem cell markers and clock genes and influence hair composition
Source: Commun Biol. 2024 Apr 12;7:453. doi: 10.1038/s42003-024-06160-2 (PMC11014975; doi:10.1038/s42003-024-06160-2)
Supplement: Supplementary file 2 — Supplementary Information [file 42003_2024_6160_MOESM2_ESM.pdf]

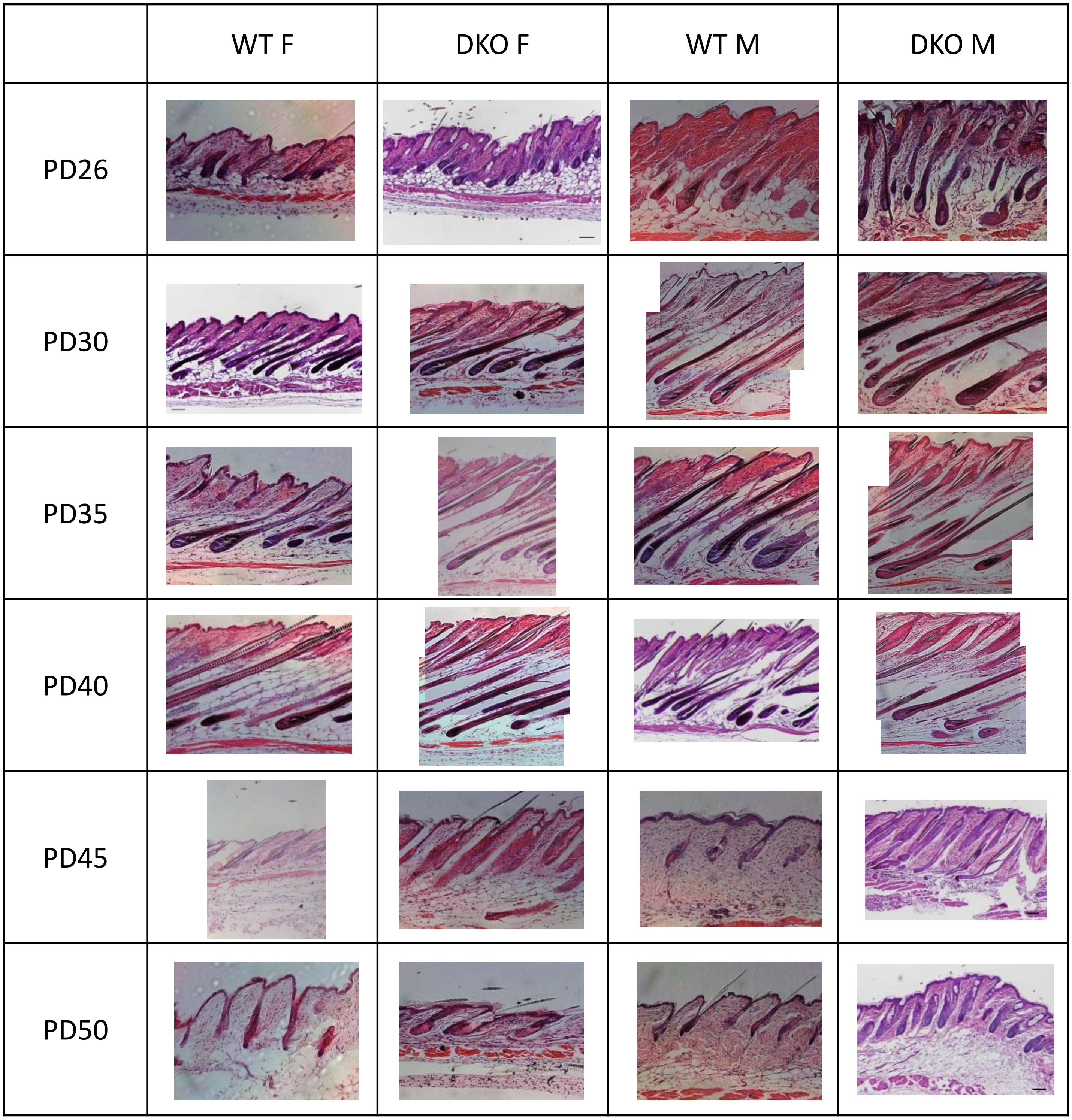

Supplementary Figure 1. Skin histology across postnatal days.
Histological images from hematoxylin and eosin staining utilized for staging of hair follicles. Taken at 10X magnification (plus magnification of the microscope).

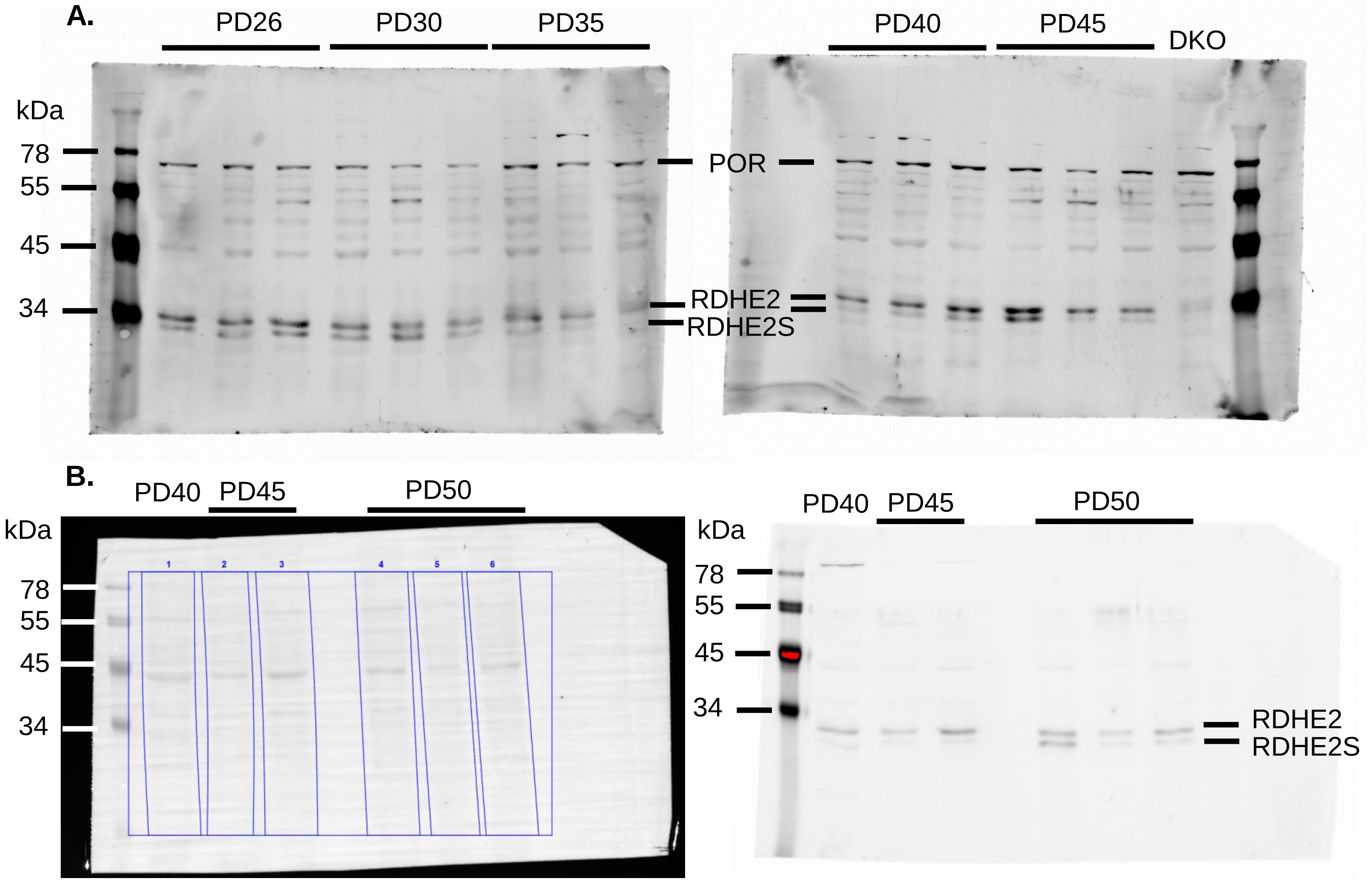

Supplementary Figure 2. RDHEs Fluctuates Across the Hair Cycle – Females.
(A) Fluorescent signal of RDHE2, RDHE2S, and POR across PDs in female WT mice with a DKO mouse skin sample for control. RDHE2/RDHE2S protein amount was normalized by POR protein amount, a microsome-specific marker. **(B)** Ponceau (left) and fluorescent signal (right) of RDHE2 and RDHE2S using remaining samples from PD40 and PD45 to compare to new samples of PD50 female skin. Ponceau stain was used due to discontinuation of previously used POR antibody.

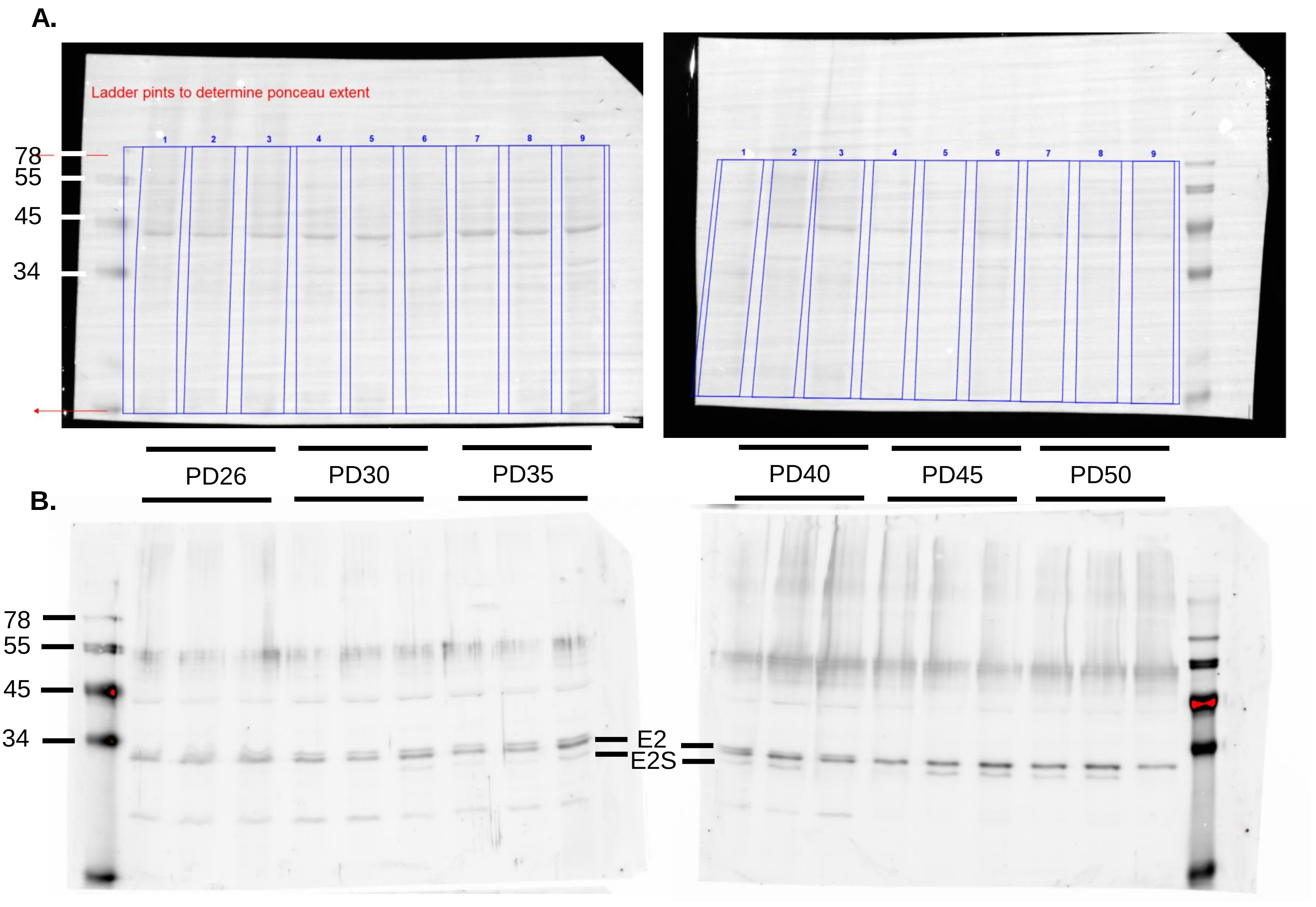

Supplementary Figure 3. RDHEs Fluctuates Across the Hair Cycle – Males.
(A) Ponceau staining of western blots of male skin microsomes across PDs. Due to the discontinuation of our POR antibody and an inability to find another suitable microsomal-specific antibody, RDHE2/RDHE2S protein amount was normalized by total protein amount using Ponceau staining. **(B)** Fluorescent imaging of RDHE2 (E2) and RDHE2S (E2S).

A. Females

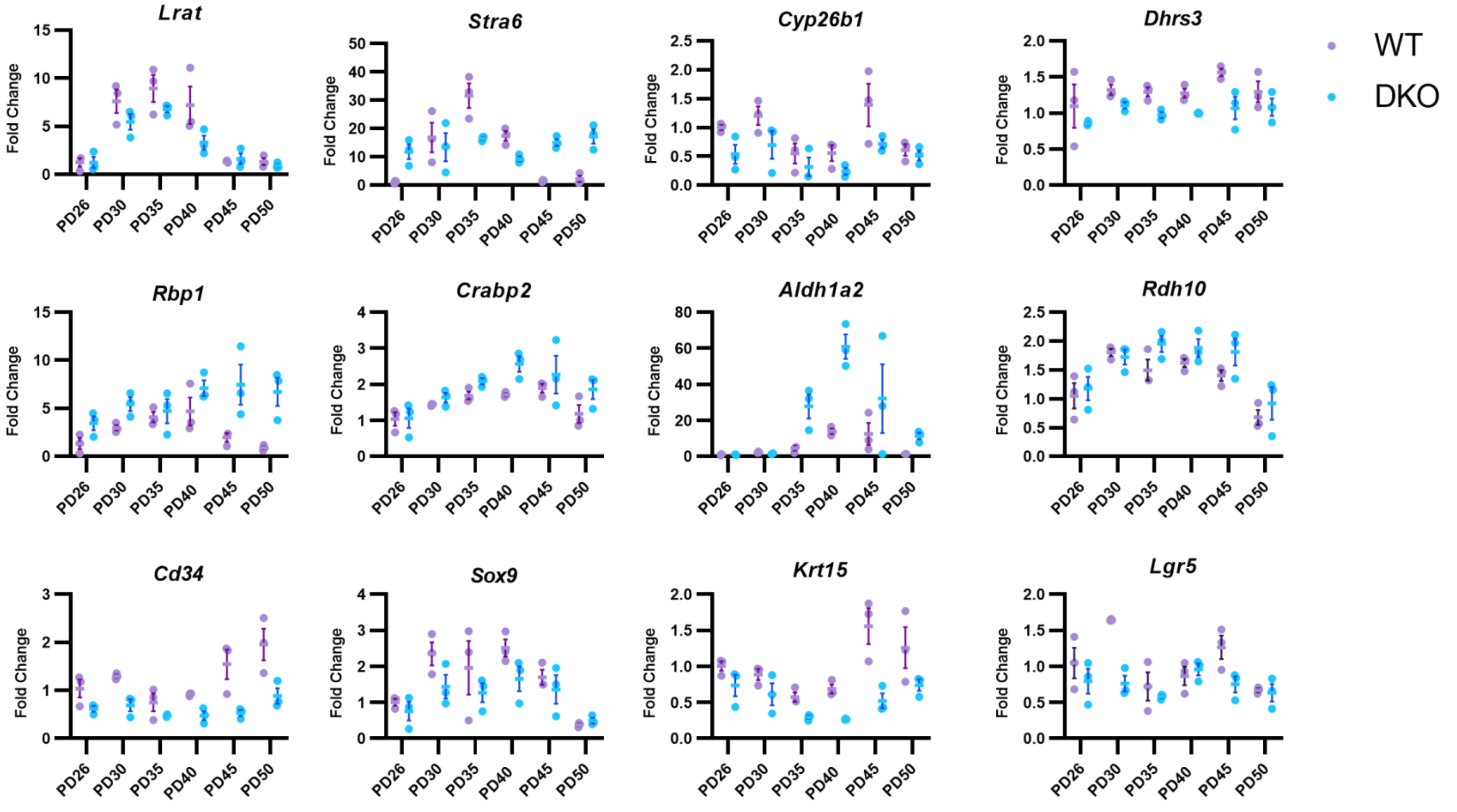

B. Males

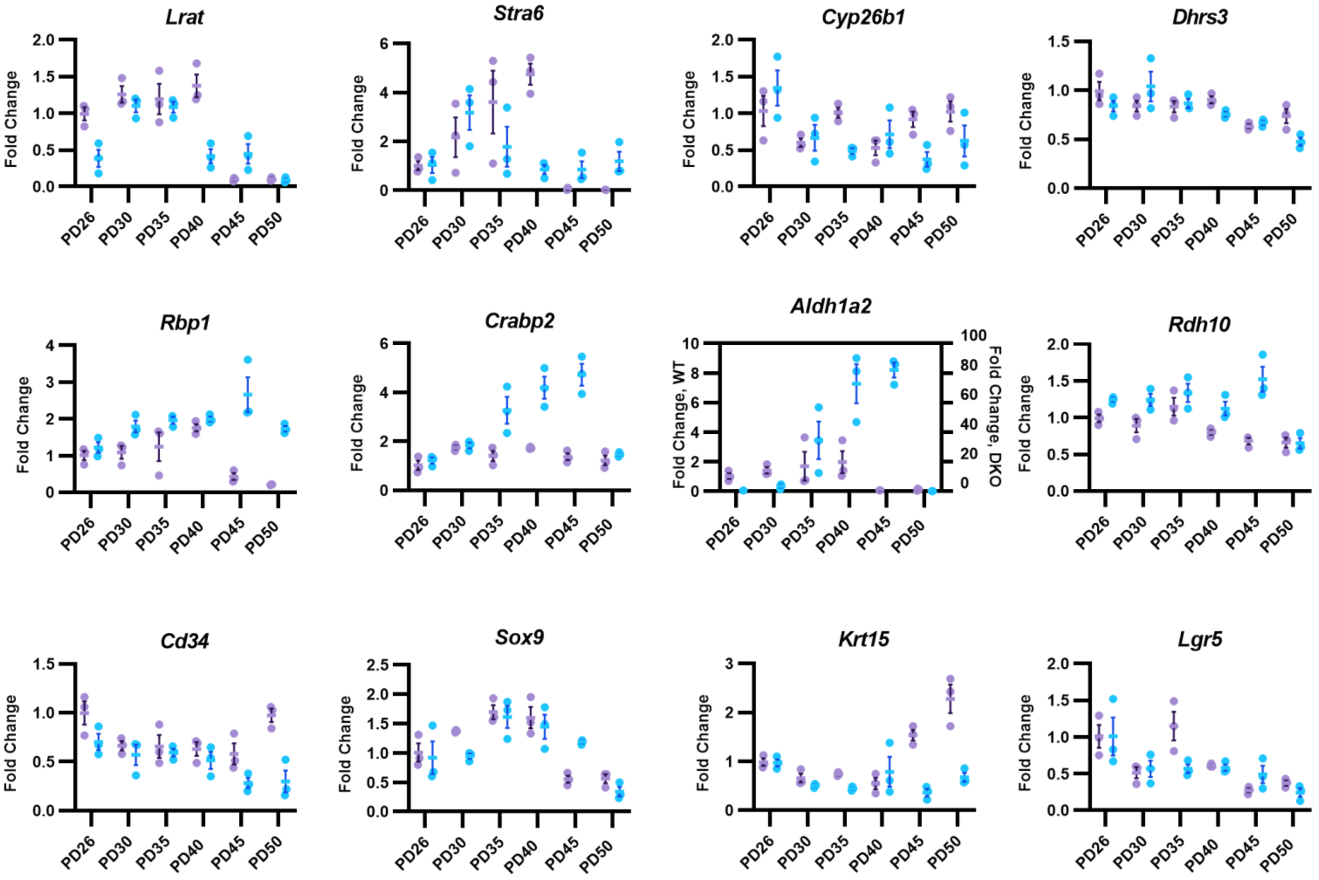

Supplementary Figure 4. Scatterplots of qPCR across PDs.
(A) Female and **(B)** male qPCR scatterplots across PDs with mean +/- SEM plotted.

Supplementary Table 1: Details of statistical analysis related to Figure 3

Fig. 3a Retinol Dehydrogenase Activity Across PDs - WT Females

Kruskal Wallis	X^2(df)	Asymptotic Sig.
	$X^2(5) = 11.830$	0.037
Pairwise Comparisons of PD	Summary	Adjusted Sig.
PD26 vs PD30	ns	> 0.999
PD26 vs PD35	ns	> 0.999
PD26 vs PD40	ns	> 0.999
PD26 vs PD45	ns	> 0.999
PD26 vs PD50	ns	0.266
PD30 vs PD35	ns	> 0.999
PD30 vs PD40	ns	> 0.999
PD30 vs PD45	ns	0.327
PD30 vs PD50	*	0.043
PD35 vs PD40	ns	> 0.999
PD35 vs PD45	ns	> 0.999
PD35 vs PD50	ns	> 0.999
PD40 vs PD45	ns	> 0.999
PD40 vs PD50	ns	> 0.999
PD45 vs PD50	ns	> 0.999

Fig. 3b Retinol Dehydrogenase Activity Across PDs - WT Males

ANOVA	F (df)	P value
Between Groups	F (5, 12) = 1.329	0.316

Fig. 3c Retinol Dehydrogenase Activity WT v DKO Females

ANOVA	F (df)	P value
Interaction	F (4, 20) = 2.081	0.121
Postnatal Day	F (4, 20) = 2.102	0.118
Genotype	F (1, 20) = 121.953	< 0.001

Fig. 3d Retinol Dehydrogenase Activity WT v DKO Males

ANOVA	F (df)	P value
Interaction	F (4, 20) = 1.022	0.420
Postnatal Day	F (4, 20) = 1.592	0.215
Genotype	F (1, 20) = 106.775	< 0.001

Fig. 3e RDHE and RDHE2S Protein Across PDs - WT Females

Kruskal Wallis	X^2(df)	Asymptotic Sig.
RDHE2	$X^2(4) = 9.500$	0.05
RDHE2S	$X^2(4) = 9.933$	0.042
Pairwise Comparisons of PD	Summary	Adjusted Sig.
PD26 vs PD30	ns	> 0.999

PD26 vs PD35	ns	> 0.999
PD26 vs PD40	ns	> 0.999
PD26 vs PD45	ns	> 0.999
PD30 vs PD35	ns	0.137
PD30 vs PD40	ns	0.081
PD30 vs PD45	ns	> 0.999
PD35 vs PD40	ns	> 0.999
PD35 vs PD45	ns	> 0.999
PD40 vs PD45	ns	> 0.999

Fig. 3f RDHE and RDHE2S Protein Across PDs - WT Males

ANOVA	F (df)	P value
RDHE2, Between Groups	F (5, 12) = 3.334	0.041
RDHE2S, Between Groups	F (5, 12) = 2.472	0.092

Multiple Comparisons, RDHE2	Summary	Significance
PD26 vs PD30	ns	0.884
PD26 vs PD35	ns	> 0.999
PD26 vs PD40	ns	0.361
PD26 vs PD45	*	0.014
PD26 vs PD50	*	0.030
PD30 vs PD35	ns	0.884
PD30 vs PD40	ns	0.439
PD30 vs PD45	*	0.019
PD30 vs PD50	*	0.039
PD35 vs PD40	ns	0.361
PD35 vs PD45	*	0.014
PD35 vs PD50	*	0.030
PD40 vs PD45	ns	0.079
PD40 vs PD50	ns	0.156
PD45 vs PD50	ns	0.693

Fig. 3g *Rdhe2* and *Rdhe2s* Across PDs - WT Females

ANOVA	F (df)	P value
RDHE2, Between Groups	F (5, 12) = 1.665	0.217
RDHE2S, Between Groups	F (5, 12) = 0.901	0.511

Fig. 3h *Rdhe2* and *Rdhe2s* Across PDs - WT Males

ANOVA	F (df)	P value
RDHE2, Between Groups	F (5, 12) = 6.812	0.003
RDHE2S, Between Groups	F (5, 12) = 0.557	0.731

Multiple Comparisons, RDHE2	Summary	Significance
PD26 vs PD30	ns	0.935
PD26 vs PD35	ns	0.978
PD26 vs PD40	ns	0.244
PD26 vs PD45	ns	0.119

PD26 vs PD50	ns	0.691
PD30 vs PD35	ns	> 0.999
PD30 vs PD40	ns	0.703
PD30 vs PD45	*	0.026
PD30 vs PD50	ns	0.236
PD35 vs PD40	ns	0.580
PD35 vs PD45	*	0.038
PD35 vs PD50	ns	0.319
PD40 vs PD45	**	0.002
PD40 vs PD50	*	0.023
PD45 vs PD50	ns	0.746

Supplementary Table 2: Details of statistical analysis related to Figure 4

Fig. 4a *Lrat*

ANOVA	F (df)	P value
Interaction	F (5, 24) = 1.633	0.189
Postnatal Day	F (5, 24) = 22.646	< 0.001
Genotype	F (1, 24) = 7.162	0.013
Multiple Comparisons	Summary	Significance
PD26 vs PD30	***	< 0.001
PD26 vs PD35	***	< 0.001
PD26 vs PD40	**	0.002
PD26 vs PD45	ns	> 0.999
PD26 vs PD50	ns	> 0.999
PD30 vs PD35	ns	0.685
PD30 vs PD40	ns	0.700
PD30 vs PD45	***	< 0.001
PD30 vs PD50	***	< 0.001
PD35 vs PD40	ns	0.073
PD35 vs PD45	***	< 0.001
PD35 vs PD50	***	< 0.001
PD40 vs PD45	**	0.003
PD40 vs PD50	**	0.001
PD45 vs PD50	ns	0.999

Fig. 4a C*yp26b1*

ANOVA	F (df)	P value
Interaction	F (5, 24) = 0.747	0.596
Postnatal Day	F (5, 24) = 5.095	0.003
Genotype	F (1, 24) = 14.894	0.001
Multiple Comparisons	Summary	Significance
PD26 vs PD30	ns	0.893
PD26 vs PD35	ns	0.385
PD26 vs PD40	ns	0.289
PD26 vs PD45	ns	0.573
PD26 vs PD50	ns	0.832
PD30 vs PD35	ns	0.057
PD30 vs PD40	*	0.037
PD30 vs PD45	ns	0.990
PD30 vs PD50	ns	0.249
PD35 vs PD40	ns	> 0.999
PD35 vs PD45	*	0.015
PD35 vs PD50	ns	0.970
PD40 vs PD45	*	0.010
PD40 vs PD50	ns	0.925
PD45 vs PD50	ns	0.082

Fig. 4a *Rbp6*

ANOVA	F (df)	P value
Interaction	F (5, 24) = 0.902	0.496
Postnatal Day	F (5, 24) = 6.667	0.001
Genotype	F (1, 24) = 10.379	0.004
Multiple Comparisons	Summary	Significance
PD26 vs PD30	ns	0.299
PD26 vs PD35	*	0.013
PD26 vs PD40	**	0.001
PD26 vs PD45	**	0.002
PD26 vs PD50	ns	0.336
PD30 vs PD35	ns	0.642
PD30 vs PD40	ns	0.121
PD30 vs PD45	ns	0.193
PD30 vs PD50	ns	> 0.999
PD35 vs PD40	ns	0.868
PD35 vs PD45	ns	0.951
PD35 vs PD50	ns	0.594
PD40 vs PD45	ns	> 0.999
PD40 vs PD50	ns	0.104
PD45 vs PD50	ns	0.168

Fig. 4a *Rdh10*

ANOVA	F (df)	P value
Interaction	F (5, 24) = 0.636	0.674
Postnatal Day	F (5, 24) = 11.223	< 0.001
Genotype	F (1, 24) = 5.583	0.027
Multiple Comparisons	Summary	Significance
PD26 vs PD30	*	0.010
PD26 vs PD35	*	0.018
PD26 vs PD40	*	0.013
PD26 vs PD45	ns	0.078
PD26 vs PD50	ns	0.455
PD30 vs PD35	ns	> 0.999
PD30 vs PD40	ns	> 0.999
PD30 vs PD45	ns	0.942
PD30 vs PD50	***	< 0.001
PD35 vs PD40	ns	> 0.999
PD35 vs PD45	ns	0.983
PD35 vs PD50	***	< 0.001
PD40 vs PD45	ns	0.960
PD40 vs PD50	***	< 0.001
PD45 vs PD50	**	0.001

Fig. 4a *Stra6*

ANOVA	F (df)	P value
Interaction	F (5, 24) = 10.524	< 0.001
Postnatal Day	F (5, 24) = 10.589	< 0.001
Genotype	F (1, 24) =1.744	0.199
Univariate Tests, GT	F (df)	Significance
PD26, WT vs DKO	F (1, 24) = 7.427	0.012
PD30, WT vs DKO	F (1, 24) = 0.770	0.389
PD35, WT vs DKO	F (1, 24) = 14.827	0.001
PD40, WT vs DKO	F (1, 24) = 4.387	0.047
PD45, WT vs DKO	F (1, 24) = 12.091	0.002
PD50, WT vs DKO	F (1, 24) = 14.862	0.001
Univariate Tests, PD	F (df)	Significance
WT	F (5, 24) = 19.908	< 0.001
DKO	F (5, 24) = 1.205	0.337
Pairwise Comparisons, PD	Summary	Significance
WT, PD26 vs PD30	**	0.007
WT, PD26 vs PD35	***	< 0.001
WT, PD26 vs PD40	**	0.005
WT, PD26 vs PD45	ns	> 0.999
WT, PD26 vs PD50	ns	> 0.999
WT, PD30 vs PD35	*	0.014
WT, PD30 vs PD40	ns	> 0.999
WT, PD30 vs PD45	*	0.010
WT, PD30 vs PD50	*	0.015
WT, PD35 vs PD40	*	0.020
WT, PD35 vs PD45	***	< 0.001
WT, PD35 vs PD50	***	< 0.001
WT, PD40 vs PD45	**	0.007
WT, PD40 vs PD50	*	0.010
WT, PD45 vs PD50	ns	> 0.999

Fig. 4a *Aldh1a2*

ANOVA	F (df)	P value
Interaction	F (5, 24) = 3.858	0.010
Postnatal Day	F (5, 24) = 9.624	< 0.001
Genotype	F (1, 24) = 19.710	< 0.001
Univariate Tests, GT	F (df)	Significance
PD26, WT vs DKO	F (1, 24) = 0.000	0.992
PD30, WT vs DKO	F (1, 24) = 0.006	0.939
PD35, WT vs DKO	F (1, 24) = 6.488	0.018
PD40, WT vs DKO	F (1, 24) = 26.656	< 0.001
PD45, WT vs DKO	F (1, 24) = 4.650	0.041

PD50, WT vs DKO	F (1, 24) = 1.200	0.284

Univariate Tests, PD	F (df)	Significance
WT	F (5, 24) = 0.808	0.555
DKO	F (5, 24) = 12.673	< 0.001

Pairwise Comparisons, PD	Summary	Significance
DKO, PD26 vs PD30	ns	> 0.999
DKO, PD26 vs PD35	ns	0.102
DKO, PD26 vs PD40	***	< 0.001
DKO, PD26 vs PD45	*	0.033
DKO, PD26 vs PD50	ns	0.991
DKO, PD30 vs PD35	ns	0.118
DKO, PD30 vs PD40	***	< 0.001
DKO, PD30 vs PD45	*	0.039
DKO, PD30 vs PD50	ns	0.995
DKO, PD35 vs PD40	*	0.019
DKO, PD35 vs PD45	ns	> 0.999
DKO, PD35 vs PD50	ns	0.729
DKO, PD40 vs PD45	ns	0.06
DKO, PD40 vs PD50	***	< 0.001
DKO, PD45 vs PD50	ns	0.381

Fig. 4a *Rbp1*

ANOVA	F (df)	P value
Interaction	F (5, 24) = 1.961	0.121
Postnatal Day	F (5, 24) = 2.480	0.060
Genotype	F (1, 24) = 27.963	< 0.001

Fig. 4a *Dhrs3*

ANOVA	F (df)	P value
Interaction	F (5, 24) = 0.400	0.844
Postnatal Day	F (5, 24) = 1.814	0.148
Genotype	F (1, 24) = 18.269	< 0.001

Fig. 4b *Lrat*

ANOVA	F (df)	P value
Interaction	F (5, 24) = 9.119	< 0.001
Postnatal Day	F (5, 24) = 34.457	< 0.001
Genotype	F (1, 24) = 15.797	0.001

Univariate Tests, GT	F (df)	Significance
PD26, WT vs DKO	F (1, 24) = 15.665	0.001
PD30, WT vs DKO	F (1, 24) = 0.995	0.329
PD35, WT vs DKO	F (1, 24) = 0.514	0.480
PD40, WT vs DKO	F (1, 24) = 38.993	< 0.001
PD45, WT vs DKO	F (1, 24) = 5.217	0.032

PD50, WT vs DKO	F (1, 24) = 0.011		0.919
Univariate Tests, PD	F (df)	Significance	
WT	F (5, 24) = 29.044		< 0.001
DKO	F (5, 24) = 14.532		< 0.001
Pairwise Comparisons, PD	Summary	Significance	
WT, PD26 vs PD30	ns		0.790
WT, PD26 vs PD35	ns		0.963
WT, PD26 vs PD40	ns		0.262
WT, PD26 vs PD45	***		< 0.001
WT, PD26 vs PD50	***		< 0.001
WT, PD30 vs PD35	ns		> 0.999
WT, PD30 vs PD40	ns		> 0.999
WT, PD30 vs PD45	***		< 0.001
WT, PD30 vs PD50	***		< 0.001
WT, PD35 vs PD40	ns		0.988
WT, PD35 vs PD45	***		< 0.001
WT, PD35 vs PD50	***		< 0.001
WT, PD40 vs PD45	***		< 0.001
WT, PD40 vs PD50	***		< 0.001
WT, PD45 vs PD50	ns		> 0.999
DKO, PD26 vs PD30	**		0.001
DKO, PD26 vs PD35	**		0.002
DKO, PD26 vs PD40	ns		> 0.999
DKO, PD26 vs PD45	ns		> 0.999
DKO, PD26 vs PD50	ns		0.629
DKO, PD30 vs PD35	ns		> 0.999
DKO, PD30 vs PD40	**		0.002
DKO, PD30 vs PD45	**		0.004
DKO, PD30 vs PD50	***		< 0.001
DKO, PD35 vs PD40	**		0.003
DKO, PD35 vs PD45	**		0.005
DKO, PD35 vs PD50	***		< 0.001
DKO, PD40 vs PD45	ns		> 0.999
DKO, PD40 vs PD50	ns		0.477
DKO, PD45 vs PD50	ns		0.357

Fig. 4b *Cyp26b1*

ANOVA	F (df)	P value	
Interaction	F (5, 24) = 3.223		0.023
Postnatal Day	F (5, 24) = 4.156		0.007
Genotype	F (1, 24) = 2.969		0.098
Univariate Tests, GT	F (df)	Significance	
PD26, WT vs DKO	F (1, 24) = 2.195		0.151
PD30, WT vs DKO	F (1, 24) = 0.103		0.751

PD35, WT vs DKO	F (1, 24) = 6.038	0.022
PD40, WT vs DKO	F (1, 24) = 0.741	0.398
PD45, WT vs DKO	F (1, 24) = 6.443	0.018
PD50, WT vs DKO	F (1, 24) = 3.567	0.071

Univariate Tests, PD	F (df)	Significance
WT	F (5, 24) = 2.281	0.079
DKO	F (5, 24) = 5.098	0.003

Pairwise Comparisons, PD	Summary	Significance
DKO, PD26 vs PD30	ns	0.057
DKO, PD26 vs PD35	**	0.007
DKO, PD26 vs PD40	ns	0.092
DKO, PD26 vs PD45	**	0.002
DKO, PD26 vs PD50	*	0.034
DKO, PD30 vs PD35	ns	0.999
DKO, PD30 vs PD40	ns	> 0.999
DKO, PD30 vs PD45	sn	0.951
DKO, PD30 vs PD50	ns	> 0.999
DKO, PD35 vs PD40	ns	0.995
DKO, PD35 vs PD45	ns	> 0.999
DKO, PD35 vs PD50	ns	> 0.999
DKO, PD40 vs PD45	ns	0.871
DKO, PD40 vs PD50	ns	> 0.999
DKO, PD45 vs PD50	ns	0.989

Fig. 4b *Rbp6*

ANOVA	F (df)	P value
Interaction	F (5, 24) = 13.323	< 0.001
Postnatal Day	F (5, 24) = 18.257	< 0.001
Genotype	F (1, 24) = 77.068	< 0.001

Univariate Tests, GT	F (df)	Significance
PD26, WT vs DKO	F (1, 24) = 0.269	0.609
PD30, WT vs DKO	F (1, 24) = 0.052	0.822
PD35, WT vs DKO	F (1, 24) = 23.836	< 0.001
PD40, WT vs DKO	F (1, 24) = 41.891	< 0.001
PD45, WT vs DKO	F (1, 24) = 77.257	< 0.001
PD50, WT vs DKO	F (1, 24) = 0.376	0.546

Univariate Tests, PD	F (df)	Significance
WT	F (5, 24) = 1.109	0.382
DKO	F (5, 24) = 30.471	< 0.001

Pairwise Comparisons, PD	Summary	Significance
DKO, PD26 vs PD30	ns	0.844
DKO, PD26 vs PD35	***	< 0.001

DKO, PD26 vs PD40	***	< 0.001
DKO, PD26 vs PD45	***	< 0.001
DKO, PD26 vs PD50	ns	> 0.999
DKO, PD30 vs PD35	*	0.014
DKO, PD30 vs PD40	***	< 0.001
DKO, PD30 vs PD45	***	< 0.001
DKO, PD30 vs PD50	ns	0.998
DKO, PD35 vs PD40	ns	0.299
DKO, PD35 vs PD45	*	0.013
DKO, PD35 vs PD50	**	0.001
DKO, PD40 vs PD45	ns	0.95
DKO, PD40 vs PD50	***	< 0.001
DKO, PD45 vs PD50	***	< 0.001

Fig. 4b *Rdh10*

ANOVA	F (df)	P value
Interaction	F (5, 24) = 4.936	0.003
Postnatal Day	F (5, 24) = 10.049	< 0.001
Genotype	F (1, 24) = 39.585	< 0.001
Univariate Tests, GT	F (df)	Significance
PD26, WT vs DKO	F (1, 24) = 0.269	0.609
PD30, WT vs DKO	F (1, 24) = 0.052	0.822
PD35, WT vs DKO	F (1, 24) = 23.836	< 0.001
PD40, WT vs DKO	F (1, 24) = 41.891	< 0.001
PD45, WT vs DKO	F (1, 24) = 77.257	< 0.001
PD50, WT vs DKO	F (1, 24) = 0.376	0.546
Univariate Tests, PD	F (df)	Significance
WT	F (5, 24) = 4.346	0.006
DKO	F (5, 24) = 10.639	< 0.001
Pairwise Comparisons, PD	Summary	Significance
WT, PD26 vs PD30	ns	> 0.999
WT, PD26 vs PD35	ns	0.973
WT, PD26 vs PD40	ns	0.924
WT, PD26 vs PD45	ns	0.313
WT, PD26 vs PD50	ns	0.225
WT, PD30 vs PD35	ns	0.543
WT, PD30 vs PD40	ns	> 0.999
WT, PD30 vs PD45	ns	0.849
WT, PD30 vs PD50	ns	0.739
WT, PD35 vs PD40	ns	0.159
WT, PD35 vs PD45	*	0.018
WT, PD35 vs PD50	*	0.012
WT, PD40 vs PD45	ns	0.999
WT, PD40 vs PD50	ns	0.992

WT, PD45 vs PD50	ns	> 0.999
DKO, PD26 vs PD30	ns	> 0.999
DKO, PD26 vs PD35	ns	> 0.999
DKO, PD26 vs PD40	ns	0.999
DKO, PD26 vs PD45	ns	0.450
DKO, PD26 vs PD50	**	0.002
DKO, PD30 vs PD35	ns	> 0.999
DKO, PD30 vs PD40	ns	0.999
DKO, PD30 vs PD45	ns	0.450
DKO, PD30 vs PD50	**	0.002
DKO, PD35 vs PD40	ns	0.822
DKO, PD35 vs PD45	ns	0.929
DKO, PD35 vs PD50	***	< 0.001
DKO, PD40 vs PD45	ns	0.069
DKO, PD40 vs PD50	*	0.016
DKO, PD45 vs PD50	***	< 0.001

Fig. 4b *Stra6*

ANOVA	F (df)	P value	
Interaction	F (5, 24) = 5.937		0.001
Postnatal Day	F (5, 24) = 7.469		< 0.001
Genotype	F (1, 24) = 1.818		0.190
Univariate Tests, GT	F (df)	Significance	
PD26, WT vs DKO	F (1, 24) = 0.001		0.997
PD30, WT vs DKO	F (1, 24) = 1.490		0.234
PD35, WT vs DKO	F (1, 24) = 4.860		0.037
PD40, WT vs DKO	F (1, 24) = 22.249		< 0.001
PD45, WT vs DKO	F (1, 24) = 0.923		0.346
PD50, WT vs DKO	F (1, 24) = 1.982		0.172
Univariate Tests, PD	F (df)	Significance	
WT	F (5, 24) = 11.027		< 0.001
DKO	F (5, 24) = 2.380		0.069
Pairwise Comparisons, PD	Summary	Significance	
WT, PD26 vs PD30	ns		0.943
WT, PD26 vs PD35	ns		0.064
WT, PD26 vs PD40	**		0.002
WT, PD26 vs PD45	ns		0.989
WT, PD26 vs PD50	ns		0.985
WT, PD30 vs PD35	ns		0.775
WT, PD30 vs PD40	ns		0.068
WT, PD30 vs PD45	ns		0.228
WT, PD30 vs PD50	ns		0.212
WT, PD35 vs PD40	ns		0.950
WT, PD35 vs PD45	**		0.004

WT, PD35 vs PD50	**	0.003
WT, PD40 vs PD45	***	< 0.001
WT, PD40 vs PD50	***	< 0.001
WT, PD45 vs PD50	ns	> 0.999

Fig. 4b *Aldh1a2*

ANOVA	F (df)	P value
Interaction	F (5, 24) = 22.881	< 0.001
Postnatal Day	F (5, 24) = 23.021	< 0.001
Genotype	F (1, 24) = 96.053	< 0.001
Univariate Tests, GT	F (df)	Significance
PD26, WT vs DKO	F (1, 24) = 0.004	0.949
PD30, WT vs DKO	F (1, 24) = 0.075	0.786
PD35, WT vs DKO	F (1, 24) = 17.735	< 0.001
PD40, WT vs DKO	F (1, 24) = 82.256	< 0.001
PD45, WT vs DKO	F (1, 24) = 110.391	< 0.001
PD50, WT vs DKO	F (1, 24) = 0.000	0.993
Univariate Tests, PD	F (df)	Significance
WT	F (5, 24) = 0.022	> 0.999
DKO	F (5, 24) = 45.881	< 0.001
Pairwise Comparisons, PD	Summary	Significance
DKO, PD26 vs PD30	ns	> 0.999
DKO, PD26 vs PD35	**	0.003
DKO, PD26 vs PD40	***	< 0.001
DKO, PD26 vs PD45	***	< 0.001
DKO, PD26 vs PD50	ns	> 0.999
DKO, PD30 vs PD35	**	0.008
DKO, PD30 vs PD40	***	< 0.001
DKO, PD30 vs PD45	***	< 0.001
DKO, PD30 vs PD50	ns	> 0.999
DKO, PD35 vs PD40	**	0.001
DKO, PD35 vs PD45	***	< 0.001
DKO, PD35 vs PD50	**	0.003
DKO, PD40 vs PD45	ns	0.985
DKO, PD40 vs PD50	***	< 0.001
DKO, PD45 vs PD50	***	< 0.001

Fig. 4b *Rbp1*

ANOVA	F (df)	P value
Interaction	F (5, 24) = 7.399	< 0.001
Postnatal Day	F (5, 24) = 5.219	0.002
Genotype	F (1, 24) = 62.520	< 0.001
Univariate Tests, GT	F (df)	Significance

PD26, WT vs DKO	F (1, 24) = 0.537	0.471
PD30, WT vs DKO	F (1, 24) = 5.915	0.023
PD35, WT vs DKO	F (1, 24) = 6.190	0.020
PD40, WT vs DKO	F (1, 24) = 0.652	0.427
PD45, WT vs DKO	F (1, 24) = 58.710	< 0.001
PD50, WT vs DKO	F (1, 24) = 27.514	< 0.001

Univariate Tests, PD	F (df)	Significance
WT	F (5, 24) = 7.459	< 0.001
DKO	F (5, 24) = 5.159	0.002

Pairwise Comparisons, PD	Summary	Significance
WT, PD26 vs PD30	ns	> 0.999
WT, PD26 vs PD35	ns	> 0.999
WT, PD26 vs PD40	ns	0.217
WT, PD26 vs PD45	ns	0.602
WT, PD26 vs PD50	ns	0.161
WT, PD30 vs PD35	ns	> 0.999
WT, PD30 vs PD40	ns	0.366
WT, PD30 vs PD45	ns	0.399
WT, PD30 vs PD50	ns	0.087
WT, PD35 vs PD40	ns	0.758
WT, PD35 vs PD45	ns	0.139
WT, PD35 vs PD50	*	0.024
WT, PD40 vs PD45	**	0.002
WT, PD40 vs PD50	***	< 0.001
WT, PD45 vs PD50	ns	> 0.999
DKO, PD26 vs PD30	ns	0.602
DKO, PD26 vs PD35	ns	0.221
DKO, PD26 vs PD40	ns	0.186
DKO, PD26 vs PD45	**	0.001
DKO, PD26 vs PD50	ns	0.747
DKO, PD30 vs PD35	ns	> 0.999
DKO, PD30 vs PD40	ns	> 0.999
DKO, PD30 vs PD45	ns	0.095
DKO, PD30 vs PD50	ns	> 0.999
DKO, PD35 vs PD40	ns	> 0.999
DKO, PD35 vs PD45	ns	0.326
DKO, PD35 vs PD50	ns	> 0.999
DKO, PD40 vs PD45	ns	0.378
DKO, PD40 vs PD50	ns	0.999
DKO, PD45 vs PD50	ns	0.060

Fig. 4b *Dhrs3*

ANOVA	F (df)	P value
Interaction	F (5, 24) = 3.102	0.027
Postnatal Day	F (5, 24) = 8.647	< 0.001

Genotype	F (1, 24) = 1.712		0.203
Univariate Tests, GT	F (df)	Significance	
PD26, WT vs DKO	F (1, 24) = 2.661		0.116
PD30, WT vs DKO	F (1, 24) = 4.309		0.049
PD35, WT vs DKO	F (1, 24) = 0.110		0.743
PD40, WT vs DKO	F (1, 24) = 2.476		0.129
PD45, WT vs DKO	F (1, 24) = 0.116		0.737
PD50, WT vs DKO	F (1, 24) = 7.548		0.011
Univariate Tests, PD	F (df)	Significance	
WT	F (5, 24) = 3.543		0.015
DKO	F (5, 24) = 8.205		< 0.001
Pairwise Comparisons, PD	Summary	Significance	
WT, PD26 vs PD30	ns		0.860
WT, PD26 vs PD35	ns		0.814
WT, PD26 vs PD40	ns		0.998
WT, PD26 vs PD45	*		0.013
WT, PD26 vs PD50	ns		0.163
WT, PD30 vs PD35	ns		> 0.999
WT, PD30 vs PD40	ns		> 0.999
WT, PD30 vs PD45	ns		0.438
WT, PD30 vs PD50	ns		0.991
WT, PD35 vs PD40	ns		> 0.999
WT, PD35 vs PD45	ns		0.496
WT, PD35 vs PD50	ns		0.996
WT, PD40 vs PD45	ns		0.126
WT, PD40 vs PD50	ns		0.755
WT, PD45 vs PD50	ns		0.995
DKO, PD26 vs PD30	ns		0.504
DKO, PD26 vs PD35	ns		> 0.999
DKO, PD26 vs PD40	ns		0.999
DKO, PD26 vs PD45	ns		0.715
DKO, PD26 vs PD50	*		0.012
DKO, PD30 vs PD35	ns		0.716
DKO, PD30 vs PD40	ns		0.088
DKO, PD30 vs PD45	**		0.009
DKO, PD30 vs PD50	***		< 0.001
DKO, PD35 vs PD40	ns		0.986
DKO, PD35 vs PD45	ns		0.502
DKO, PD35 vs PD50	**		0.006
DKO, PD40 vs PD45	ns		0.999
DKO, PD40 vs PD50	ns		0.101
DKO, PD45 vs PD50	ns		0.575

Supplementary Table 3: Details of statistical analysis related to Figure 5

Fig. 5a *Cd34*

ANOVA	F (df)	P value
Interaction	F (5, 24) = 1.951	0.123
Postnatal Day	F (5, 24) = 6.015	0.001
Genotype	F (1, 24) = 43.783	< 0.001
Multiple Comparisons	Summary	Significance
PD26 vs PD30	ns	0.919
PD26 vs PD35	ns	0.790
PD26 vs PD40	ns	0.969
PD26 vs PD45	ns	0.804
PD26 vs PD50	*	0.018
PD30 vs PD35	ns	0.245
PD30 vs PD40	ns	0.510
PD30 vs PD45	ns	> 0.999
PD30 vs PD50	ns	0.147
PD35 vs PD40	ns	0.995
PD35 vs PD45	ns	0.152
PD35 vs PD50	**	0.001
PD40 vs PD45	ns	0.355
PD40 vs PD50	**	0.003
PD45 vs PD50	ns	0.238

Fig. 5a *Sox9*

ANOVA	F (df)	P value
Interaction	F (5, 24) = 0.738	0.602
Postnatal Day	F (5, 24) = 7.302	< 0.001
Genotype	F (1, 24) = 6.661	0.160
Multiple Comparisons	Summary	Significance
PD26 vs PD30	ns	0.051
PD26 vs PD35	ns	0.263
PD26 vs PD40	*	0.014
PD26 vs PD45	ns	0.394
PD26 vs PD50	ns	0.749
PD30 vs PD35	ns	0.954
PD30 vs PD40	ns	0.992
PD30 vs PD45	ns	0.868
PD30 vs PD50	**	0.002
PD35 vs PD40	ns	0.711
PD35 vs PD45	ns	> 0.999
PD35 vs PD50	*	0.016
PD40 vs PD45	ns	0.548
PD40 vs PD50	**	0.001
PD45 vs PD50	*	0.03

Fig. 5a *Krt15*

ANOVA	F (df)	P value
Interaction	F (5, 24) = 2.348	0.072
Postnatal Day	F (5, 24) = 7.157	< 0.001
Genotype	F (1, 24) = 34.903	< 0.001
Multiple Comparisons	Summary	Significance
PD26 vs PD30	ns	0.949
PD26 vs PD35	*	0.041
PD26 vs PD40	ns	0.078
PD26 vs PD45	ns	0.809
PD26 vs PD50	ns	0.928
PD30 vs PD35	ns	0.232
PD30 vs PD40	ns	0.370
PD30 vs PD45	ns	0.309
PD30 vs PD50	ns	0.467
PD35 vs PD40	ns	> 0.999
PD35 vs PD45	**	0.002
PD35 vs PD50	**	0.004
PD40 vs PD45	**	0.005
PD40 vs PD50	**	0.009
PD45 vs PD50	ns	> 0.999

Fig. 5a *Lgr5*

ANOVA	F (df)	P value
Interaction	F (5, 24) = 3.744	0.012
Postnatal Day	F (5, 24) = 5.306	0.002
Genotype	F (1, 24) = 14.784	0.001
Univariate Tests, GT	F (df)	Significance
PD26, WT vs DKO	F (1, 24) = 1.832	0.188
PD30, WT vs DKO	F (1, 24) = 23.150	< 0.001
PD35, WT vs DKO	F (1, 24) = 0.556	0.463
PD40, WT vs DKO	F (1, 24) = 0.223	0.641
PD45, WT vs DKO	F (1, 24) = 7.702	0.011
PD50, WT vs DKO	F (1, 24) = 0.042	0.839
Univariate Tests, PD	F (df)	Significance
WT	F (5, 24) = 8.051	< 0.001
DKO	F (5, 24) = 0.999	0.439
Pairwise Comparisons, PD	Summary	Significance
WT, PD26 vs PD30	*	0.049
WT, PD26 vs PD35	ns	0.754
WT, PD26 vs PD40	ns	0.998
WT, PD26 vs PD45	ns	0.987

WT, PD26 vs PD50	ns	0.575
WT, PD30 vs PD35	**	0.001
WT, PD30 vs PD40	**	0.004
WT, PD30 vs PD45	ns	0.523
WT, PD30 vs PD50	***	< 0.001
WT, PD35 vs PD40	ns	> 0.999
WT, PD35 vs PD45	ns	0.100
WT, PD35 vs PD50	ns	> 0.999
WT, PD40 vs PD45	ns	0.475
WT, PD40 vs PD50	ns	0.996
WT, PD45 vs PD50	ns	0.057

Fig. 5b *Cd34*

ANOVA	F (df)	P value
Interaction	F (5, 24) = 3.418	0.018
Postnatal Day	F (5, 24) = 4.704	0.004
Genotype	F (1, 24) = 25.684	< 0.001
Univariate Tests, GT	F (df)	Significance
PD26, WT vs DKO	F (1, 24) = 5.656	0.026
PD30, WT vs DKO	F (1, 24) = 0.549	0.466
PD35, WT vs DKO	F (1, 24) = 0.265	0.611
PD40, WT vs DKO	F (1, 24) = 0.868	0.361
PD45, WT vs DKO	F (1, 24) = 5.764	0.024
PD50, WT vs DKO	F (1, 24) = 29.672	< 0.001
Univariate Tests, PD	F (df)	Significance
WT	F (5, 24) = 4.482	0.005
DKO	F (5, 24) = 3.641	0.014
Pairwise Comparisons, PD	Summary	Significance
WT, PD26 vs PD30	ns	0.170
WT, PD26 vs PD35	ns	0.153
WT, PD26 vs PD40	ns	0.093
WT, PD26 vs PD45	*	0.04
WT, PD26 vs PD50	ns	> 0.999
WT, PD30 vs PD35	ns	> 0.999
WT, PD30 vs PD40	ns	> 0.999
WT, PD30 vs PD45	ns	> 0.999
WT, PD30 vs PD50	ns	0.238
WT, PD35 vs PD40	ns	> 0.999
WT, PD35 vs PD45	ns	> 0.999
WT, PD35 vs PD50	ns	0.135
WT, PD40 vs PD45	ns	> 0.999
WT, PD40 vs PD50	ns	0.135
WT, PD45 vs PD50	ns	0.059
DKO, PD26 vs PD30	ns	0.995

DKO, PD26 vs PD35	ns	0.999
DKO, PD26 vs PD40	ns	0.893
DKO, PD26 vs PD45	*	0.038
DKO, PD26 vs PD50	ns	0.052
DKO, PD30 vs PD35	ns	> 0.999
DKO, PD30 vs PD40	ns	> 0.999
DKO, PD30 vs PD45	ns	0.372
DKO, PD30 vs PD50	ns	0.462
DKO, PD35 vs PD40	ns	> 0.999
DKO, PD35 vs PD45	ns	0.270
DKO, PD35 vs PD50	ns	0.345
DKO, PD40 vs PD45	ns	0.709
DKO, PD40 vs PD50	ns	0.801
DKO, PD45 vs PD50	ns	> 0.999

Fig. 5b *Sox9*

ANOVA	F (df)	P value
Interaction	F (5, 24) = 3.195	0.024
Postnatal Day	F (5, 24) = 19.221	< 0.001
Genotype	F (1, 24) = 0.459	0.505
Univariate Tests, GT	F (df)	Significance
PD26, WT vs DKO	F (1, 24) = 0.195	0.663
PD30, WT vs DKO	F (1, 24) = 4.341	0.048
PD35, WT vs DKO	F (1, 24) = 0.172	0.682
PD40, WT vs DKO	F (1, 24) = 0.566	0.459
PD45, WT vs DKO	F (1, 24) = 9.913	0.004
PD50, WT vs DKO	F (1, 24) = 1.247	0.275
Univariate Tests, PD	F (df)	Significance
WT	F (5, 24) = 12.390	< 0.001
DKO	F (5, 24) = 10.026	< 0.001
Pairwise Comparisons, PD	Summary	Significance
WT, PD26 vs PD30	ns	0.740
WT, PD26 vs PD35	*	0.033
WT, PD26 vs PD40	ns	0.103
WT, PD26 vs PD45	ns	0.412
WT, PD26 vs PD50	ns	0.453
WT, PD30 vs PD35	ns	0.842
WT, PD30 vs PD40	ns	0.990
WT, PD30 vs PD45	**	0.007
WT, PD30 vs PD50	**	0.009
WT, PD35 vs PD40	ns	> 0.999
WT, PD35 vs PD45	***	< 0.001
WT, PD35 vs PD50	***	< 0.001
WT, PD40 vs PD45	***	< 0.001

WT, PD40 vs PD50	***	< 0.001
WT, PD45 vs PD50	ns	> 0.999
DKO, PD26 vs PD30	ns	> 0.999
DKO, PD26 vs PD35	*	0.031
DKO, PD26 vs PD40	ns	0.201
DKO, PD26 vs PD45	ns	0.957
DKO, PD26 vs PD50	ns	0.124
DKO, PD30 vs PD35	*	0.044
DKO, PD30 vs PD40	ns	0.269
DKO, PD30 vs PD45	ns	0.984
DKO, PD30 vs PD50	ns	0.089
DKO, PD35 vs PD40	ns	> 0.999
DKO, PD35 vs PD45	ns	0.511
DKO, PD35 vs PD50	***	< 0.001
DKO, PD40 vs PD45	ns	0.974
DKO, PD40 vs PD50	***	< 0.001
DKO, PD45 vs PD50	**	0.005

Fig. 5b *Krt15*

ANOVA	F (df)	P value
Interaction	F (5, 24) = 13.422	< 0.001
Postnatal Day	F (5, 24) = 12.045	< 0.001
Genotype	F (1, 24) = 38.764	< 0.001
Univariate Tests, GT	F (df)	Significance
PD26, WT vs DKO	F (1, 24) = 0.010	0.923
PD30, WT vs DKO	F (1, 24) = 0.652	0.427
PD35, WT vs DKO	F (1, 24) = 2.211	0.150
PD40, WT vs DKO	F (1, 24) = 1.501	0.232
PD45, WT vs DKO	F (1, 24) = 35.549	< 0.001
PD50, WT vs DKO	F (1, 24) = 65.950	< 0.001
Univariate Tests, PD	F (df)	Significance
WT	F (5, 24) = 22.744	< 0.001
DKO	F (5, 24) = 2.723	0.044
Pairwise Comparisons, PD	Summary	Significance
WT, PD26 vs PD30	ns	0.829
WT, PD26 vs PD35	ns	0.977
WT, PD26 vs PD40	ns	0.418
WT, PD26 vs PD45	ns	0.146
WT, PD26 vs PD50	***	< 0.001
WT, PD30 vs PD35	ns	> 0.999
WT, PD30 vs PD40	ns	> 0.999
WT, PD30 vs PD45	**	0.003
WT, PD30 vs PD50	***	< 0.001
WT, PD35 vs PD40	ns	0.998

WT, PD35 vs PD45	**	0.007
WT, PD35 vs PD50	***	< 0.001
WT, PD40 vs PD45	**	0.001
WT, PD40 vs PD50	***	< 0.001
WT, PD45 vs PD50	*	0.014
DKO, PD26 vs PD30	ns	0.332
DKO, PD26 vs PD35	ns	0.194
DKO, PD26 vs PD40	ns	0.999
DKO, PD26 vs PD45	ns	0.073
DKO, PD26 vs PD50	ns	0.914
DKO, PD30 vs PD35	ns	> 0.999
DKO, PD30 vs PD40	ns	0.924
DKO, PD30 vs PD45	ns	> 0.999
DKO, PD30 vs PD50	ns	0.999
DKO, PD35 vs PD40	ns	0.777
DKO, PD35 vs PD45	ns	> 0.999
DKO, PD35 vs PD50	ns	0.988
DKO, PD40 vs PD45	ns	0.452
DKO, PD40 vs PD50	ns	> 0.999
DKO, PD45 vs PD50	ns	0.858

Fig. 5b *Lgr5*

ANOVA	F (df)	P value
Interaction	F (5, 24) = 2.494	0.059
Postnatal Day	F (5, 24) = 9.976	< 0.001
Genotype	F (1, 24) = 1.293	0.267
Multiple Comparisons	Summary	Significance
PD26 vs PD30	**	0.009
PD26 vs PD35	ns	0.821
PD26 vs PD40	*	0.028
PD26 vs PD45	***	< 0.001
PD26 vs PD50	***	< 0.001
PD30 vs PD35	ns	0.131
PD30 vs PD40	ns	0.996
PD30 vs PD45	ns	0.773
PD30 vs PD50	ns	0.420
PD35 vs PD40	ns	0.304
PD35 vs PD45	**	0.007
PD35 vs PD50	**	0.002
PD40 vs PD45	ns	0.483
PD40 vs PD50	ns	0.198
PD45 vs PD50	ns	0.991

Supplementary Table 4: Details of statistical analysis related to Figure 6

Fig. 6c Pelage Hair Types		
T-Test	t (df)	P value
Guard	t (10) = 0.839	0.421
Awl	t (10) = -3.282	0.008
Auchene	t (10) = 0.918	0.380
Zigzag	t (10) = 0.718	0.489

Supplementary Table 5: Details of statistical analysis related to Figure 7

Fig. 7a Female Clock Genes

T-Test	t (df)	P value
Dbp	t (4) = 3.076	0.037
Npas2	t (4) = 3.186	0.033
Nr1d1	t (4) = 3.138	0.035
Per2	t (4) = 1.093	0.336
Cry2	t (4) = 1.777	0.150
Clock	t (4) = 2.420	0.072

Fig. 7b Male Clock Genes

T-Test	t (df)	P value
Dbp	t (4) = 4.703	0.009
Npas2	t (4) = 2.918	0.043
Nr1d1	t (4) = 1.483	0.212
Per2	t (4) = 5.298	0.006
Cry2	t (4) = 10.803	< 0.001
Clock	t (4) = 1.772	0.151

Fig. 7c *Notch1* for WT and DKO Females

ANOVA	F (df)	P value
Interaction	F (5, 24) = 1.065	0.404
Postnatal Day	F (5, 24) = 8.116	< 0.001
Genotype	F (1, 24) = 4.587	0.043
Multiple Comparisons	Summary	Significance
PD26 vs PD30	**	0.002
PD26 vs PD35	***	< 0.001
PD26 vs PD40	**	0.007
PD26 vs PD45	*	0.024
PD26 vs PD50	ns	0.835
PD30 vs PD35	ns	0.977
PD30 vs PD40	ns	0.995
PD30 vs PD45	ns	0.901
PD30 vs PD50	*	0.034
PD35 vs PD40	ns	0.813
PD35 vs PD45	ns	0.509
PD35 vs PD50	**	0.006
PD40 vs PD45	ns	0.995
PD40 vs PD50	ns	0.100
PD45 vs PD50	ns	0.259

Fig. 7c *Notch1* for WT and DKO Males

ANOVA	F (df)	P value
Interaction	F (5, 24) = 1.635	0.189
Postnatal Day	F (5, 24) = 5.570	0.002

Genotype	F (1, 24) = 2.559	0.123
Multiple Comparisons	Summary	Significance
PD26 vs PD30	ns	0.999
PD26 vs PD35	ns	0.825
PD26 vs PD40	ns	> 0.999
PD26 vs PD45	*	0.048
PD26 vs PD50	ns	0.177
PD30 vs PD35	ns	0.602
PD30 vs PD40	ns	0.099
PD30 vs PD45	ns	0.107
PD30 vs PD50	ns	0.337
PD35 vs PD40	ns	0.901
PD35 vs PD45	**	0.003
PD35 vs PD50	*	0.013
PD40 vs PD45	*	0.032
PD40 vs PD50	ns	0.126
PD45 vs PD50	ns	0.985
