# Supplementary material for: Epidermal retinol dehydrogenases cyclically regulate stem cell markers and clock genes and influence hair composition
Source: Commun Biol. 2024 Apr 12;7:453. doi: 10.1038/s42003-024-06160-2 (PMC11014975; doi:10.1038/s42003-024-06160-2)
Supplement: Supplementary file 3 — Description of Additional Supplementary Files [file 42003_2024_6160_MOESM3_ESM.pdf]

Description of Additional Supplementary Files

File name: Supplementary Data

Description: The source data behind Figures 2-7 in the paper.
